# Localization of Oxidized Low-Density Lipoprotein and Its Relation to Plaque Morphology in Human Coronary Artery

**DOI:** 10.1371/journal.pone.0055188

**Published:** 2013-02-05

**Authors:** Yasumi Uchida, Yuko Maezawa, Yasuto Uchida, Nobuyuki Hiruta, Ei Shimoyama, Seiji Kawai

**Affiliations:** 1 Japan Foundation for Cardiovascular Research, Funabashi, Japan; 2 Department of Cellular Therapy, Chiba University, Chiba, Japan; 3 Department of Cardiology, Toho University Ohmori Hospital, Tokyo, Japan; 4 Department of Pathology, Toho University Sakura Hospital, Sakura, Japan; 5 Department of Pathology, Funabashi-Futawa Hospital, Funabashi, Japan; 6 Department of Surgery, Chiba-Kensei Hospital, Chiba, Japan; University of Virginia Health System, United States of America

## Abstract

**Objectives:**

Oxidized low-density lipoprotein (oxLDL) plays a key role in the formation of atherosclerotic plaques. However, its localization in human coronary arterial wall is not well understood. The present study was performed to visualize deposition sites and patterns of native oxLDL and their relation to plaque morphology in human coronary artery.

**Methods:**

Evans blue dye (EB) elicits a violet fluorescence by excitation at 345-nm and emission at 420-nm, and a reddish-brown fluorescence by excitation at 470-nm and emission at 515-nm characteristic of oxLDL only. Therefore, native oxLDL in excised human coronary artery were investigated by color fluorescent microscopy (CFM) using EB as a biomarker.

**Results:**

(1) By luminal surface scan with CFM, the % incidence of oxLDL in 38 normal segments, 41 white plaques and 32 yellow plaques that were classified by conventional angioscopy, was respectively 26, 44 and 94, indicating significantly (p<0.05) higher incidence in the latter than the former two groups. Distribution pattern was classified as patchy, diffuse and web-like. Web-like pattern was observed only in yellow plaques with necrotic core. (2) By transected surface scan, oxLDL deposited within superficial layer in normal segments and diffusely within both superficial and deep layers in white and yellow plaques. In yellow plaques with necrotic core, oxLDL deposited not only in the marginal zone of the necrotic core but also in the fibrous cap.

**Conclusion:**

Taken into consideration of the well-known process of coronary plaque growth, the results suggest that oxLDL begins to deposit in human coronary artery wall before plaque formation and increasingly deposits with plaque growth, exhibiting different deposition sites and patterns depending on morphological changes.

## Introduction

Oxidized low-density lipoprotein (oxLDL) plays a key role in the initiation, progression and destabilization of atherosclerotic plaques [1]–[3]. It accelerates the migration of monocytes into the vascular wall, as well as the proliferation of macrophages [4], [5], which in turn accumulate lipids, such as cholesteryl esters and oxLDL, within themselves and become foam cells [6]–[8]. In addition, they cause loss of collagen fibers by collagen-degrading enzymes such as metalloproteinases and death of collagen-synthesizing smooth muscle cells [3], [9], thus making the plaque vulnerable. The death of macrophages and foam cells results in the formation of a lipid core [10].

Trafficking of oxLDL has been performed using radiotracers such as ^125^I-MDA2, ^99^mTc-MDA2, and ^99^mTc-labeled anti-LOX-1 mlgG [11], [12], and imaging of native oxLDL has been performed using labeled antibody in animal aortas or fish vessels [13]–[15], immunohistochemically in human carotid plaques obtained by atherectomy [16], [17], or by spectroscopy in the murine aorta [18].

Imaging of native oxLDL in human coronary artery was performed immunohistochemically [19], [20]. However, its distribution sites and patterns, and their relation to plaque morphology were not fully examined.

We succeeded in two-dimensional imaging of oxLDL in human coronary plaques by means of a color fluorescent angioscopy (CFA) system using Evans blue dye (EB) as a biomarker of oxLDL, both in vitro and in vivo [21]–[26]. However, localization and deposition patters of native oxLDL within human coronary plaques and their relation to coronary plaque morphology remained obscure. In the present fluorescent microscopic study using EB as a biomarker, was performed to clarify them, and it was revealed that localization and deposition patterns of native oxLDL are closely related to plaque morphology in excised human coronary artery.

## Methods

### 1. Investigating OxLDL by Color Fluorescent Microscopy (CFM)

The color fluorescence of oxLDL and other major substances that constitute atherosclerotic plaques (Table 1) [27] was examined by means of CFM by excitation at 345-nm and emission at 420-nm (“A”-imaging), and by excitation at 470-nm and emission at 520-nm (“B”-imaging) using Evans blue dye (EB) as a biomarker. The details of CFM are described elsewhere [25].

**Table 1:** Fluorescent Colors of the Major Substances That Comprise Atherosclerotic Plaques Excited by Evans Blue Dye (EB).

Substances	Color fluorescent microscopy			
	Autofluorescence		Fluorescence in the presence of EB(10^−5^M)	
	“A”-imaging	“B”-imaging	“A”-imaging	“B”-imaging
Oxidized low-density lipoprotein	no	No	V	RB
Low-density lipoprotein	no	No	No	R
Very low-density lipoprotein	no	No	No	R
High-density lipoprotein	no	No	R	R
Lysophosphatidylcholine	no	No	R	R
Phosphatidylcholine	no	No	No	O
Triglyceride	no	No	No	no
Apolipoprotein B-100	no	No	R	R
Apolipoprotein A-1	no	No	No	no
Apolipoprotein E-2	no	No	No	no
Matrix metalloproteinase-9	no	No	No	no
Cholesterol	W	LY	No	O
Cholesteryl oleate	no	No	B	G
Cholesteryl linoreate	no	No	No	no
7-Keto-cholesterol	no	No	No	no
Oleic acid	no	No	No	no
Linoleic acid	no	No	No	no
Collagen I	B	G	B	DG
Collagen IV	LB	G	No	G
Collagens III, V	no	No	No	no
Heparan sulfate	no	No	No	no
Hyaluronic acid	no	No	No	no
Albumin	no	No	No	no
Globulins	no	No	No	no
Elastin	W	LY	R	no
Ceramide	P	Y	P	O
Hydroxyapatite	no	No	No	no
Calcium phosphate tribasic	W	LY	W∼B	LY
β-Carotene	O	O	No	no

“A”-imaging = band-pass filter (BPF) 345-nm and band-absorption filter (BAF) 420-nm. “B”-imaging = BPF 470-nm and BAF 515-nm.

B = blue. G = green. LB = light blue. LY: light yellow. O = orange. P = purple. R = red. RB = reddish-brown. V = violet. W = white. Y = yellow.

Bold characters = strong fluorescence. Non-bold characters = weak fluorescence. no = no fluorescence.

This table shows fluorescence colors of the major substances that comprise atherosclerotic plaques excited by Evans blue dye (EB). EB 10^−5 ^M was added to each substance to evoke color fluorescence. A violet fluorescence at “A”-imaging and a reddish-brown fluorescence at “B”-imaging were evoked by adding EB to oxLDL. This combination of fluorescence colors was not evoked in any other known substances that comprising atherosclerotic plaques listed in this table, indicating that this combination is characteristic of only oxLDL.

The intensity of fluorescence was categorized as strong, weak or absent when the exposure time required for imaging was taken to be within 1 s, >1 and ≤5 s, and >5 s, respectively.

In the present study, EB (Wako Co., Osaka, Japan) was diluted in distilled water to a concentration of 10^−5^ M (the maximum concentration that does not precipitate) at 37°C and then mixed with each of the major substances in atherosclerotic plaques. The evoked fluorescence was photographed at ×40.

### 2. Classification of Coronary Plaques by Conventional Angioscopy

In the present study, conventional angioscopy using white light as a light source was used to classify the coronary plaques and normal segments because their angioscopic appearances represent their macroscopic pathological changes, and this technology is widely used clinically for detection of vulnerable coronary plaques [28]. The details of the system are described elsewhere [21].

Plaque is defined as a nonmobile, protuberant or lining mass that is clearly demarcated from the adjacent normal wall and the shape, location and color of which do not alter under the influence of a saline solution flush. Plaque is further classified as white or yellow based on its surface color. A normal segment is defined as milky-white and smooth-surfaced without any protrusions [29].

Images of the plaque obtained by conventional angioscopy were classified as white or yellow using an AquaCosmos image analyzer (C7746, Hamamatsu Photonics), which sets a window on an appropriate portion of an image and separates the color within the window into three primary colors, namely red, green and blue. Plaque was defined as “white” when the intensity ratio of red : green : blue was 1.0∶ 0.9∼1.1∶ 0.9∼1.1, respectively, and as “yellow” when it was 1.0∶ 0.8∼1.2∶ 0.3∼0.6, respectively [25].

### 3. Observation of Coronary Plaques by Conventional Angioscopy

#### Ethics statement

The study was carried out with the approval of the Ethical Committees of the Japan Foundation for Cardiovascular Research, Ethical Committee of Toho University, Ethical Committee of Funabashi-Futawa Hospital, Ethical Committee of Chiba-kensei Hospital, and after obtaining written informed consent from the families concerned on the use of excised coronary arteries for pathological study to clarify mechanisms of coronary atherosclerosis.

Fifty-five coronary arteries (19 left anterior descending arteries, 18 left circumflex arteries, 18 right coronary arteries) were detached from 19 cadavers [61±3 years old, 7 females, 12 males; cause of death: acute myocardial infarction (4), chronic renal disease (4), hepatocellular carcinoma (3) cerebral infarction (2), cerebral bleeding (2), gastric cancer (1), pancreatic cancer (1), subarachnoid hemorrhage (1), aortic dissection (1)].

A Y-connector was introduced into the proximal portion of each coronary artery for perfusion with saline solution at a rate of 10 mL/min and then the angioscope was introduced through the connector into the artery for observation. Initially, conventional angioscopy was carried out to detect plaque and because the light from the angioscope’s tip was visible through the coronary wall, the site of the targeted plaque could be confirmed.

### 4. Imaging of OxLDL by CFM

A total of 150 coronary segments which contained plaques and 79 normal segments were isolated by transecting their proximal and distal ends along the shorter axes to avoid any damage to the plaque. The isolated segment was then cut longitudinally to open the lumen.

#### Scanning the luminal surface

The 40 white plaques, 33 yellow plaques and 38 normal segments were mounted on deck glass in such a way that the luminal surface faced the deck glass. The surface was then scanned by CFM to investigate the fluorescence of the substances listed in Table 1 at ×40 or ×10 using similar light wavelength filters as were used for examination of fluorescence of the substances that comprising atherosclerotic plaques.

#### Scanning the transected surface

In the remaining 42 white plaques, 35 yellow plaques and 41 normal segments, the center of each plaque or segment was transected and one half was mounted on a deck glass such that the transected surface faced the glass. After obtaining control picture, 0.1 mL EB solution was dripped onto the specimen. By this maneuver, the EB solution diffused into the transected surface and stained oxLDL.

The plaques were divided into two layers, namely superficial (≤ 200-µm from the luminal surface) and deep (>200-µm from the luminal surface), and relationships between the deposition site of oxLDL and plaque morphology studied by conventional angioscopy were examined.

#### Definition of fluorescent color

The fluorescent color, that was elicited by EB, was analyzed by AquaCosmos image analyzer. The fluorescent color obtained by CFM was divided into red, green and blue, and the fluorescent color was defined as “violet” when the ratio of red, green, and blue was 1.0∶0.65∼0.85∶0.75∼0.95, respectively, and it was defined as reddish-brown when it was 1.0∶0.35∼0.55∶0.20∼0.30, respectively.

### 5. Histology

After CFA and CFM scanning, each sample was cut into slices along the shorter axis and stained with Oil Red O and methylene blue (MB), which stain lipids red, calcium black, and collagen fibers and smooth muscles blue [25], [29]. In a preliminary study, cholesterol, cholesteryl esters and ceramide stained red with Oil Red O, but not any of the other substances, including apolipoproteins and lipoproteins, listed in Table 1.

### 6. Statistical Analysis

The data obtained were tested by χ^2^ formulae. A value of p<0.05 was considered to be statistically significant. The differences in % incidence, deposition sites and patterns oxLDL were compared among normal segments, white plaques and yellow plaques.

## Results

### 1. Fluorescence Color of OxLDL Excited by EB

**Figure 1:**
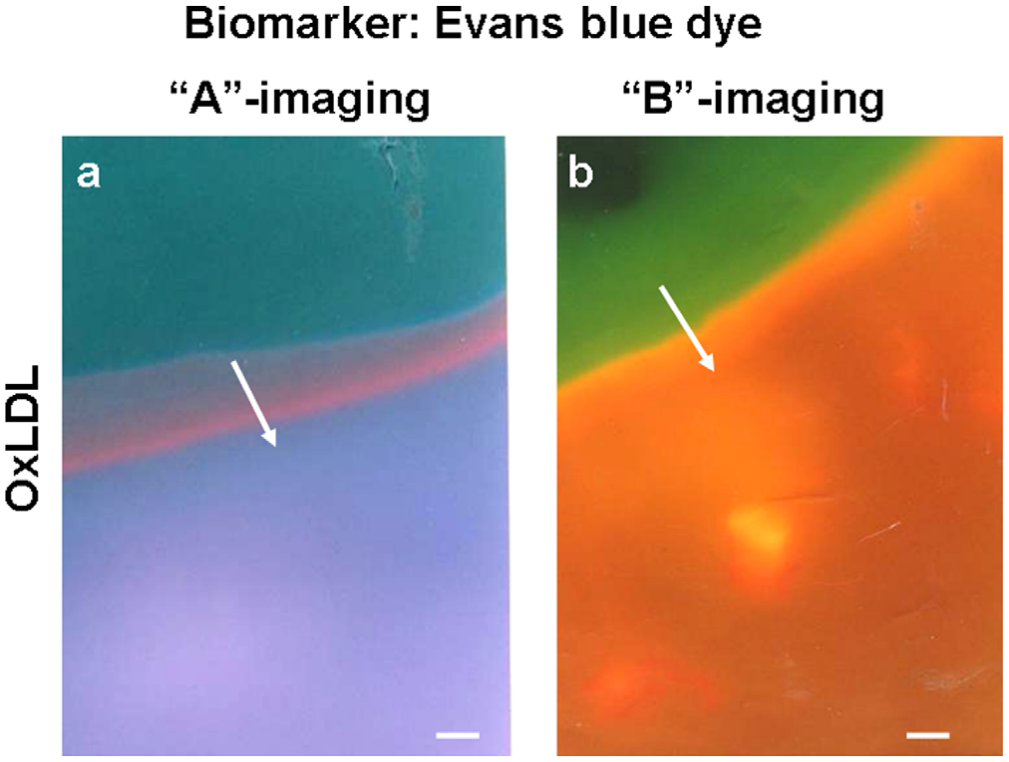
Fluorescence of oxidized low-density lipoprotein (oxLDL). OxLDL fluoresced violet with “A”-imaging (a) and reddish-brown with “B”-imaging (b) when excited in the presence of 10^–5^ M Evans blue (EB) dye. Bar = 100 - µm.

In the presence of EB, oxLDL exhibited a violet fluorescence with “A”-imaging and a reddish-brown fluorescence with “B”-imaging (Fig. 1). This combination of fluorescence colors was not exhibited by any of the other major known substances that comprise atherosclerotic plaques, indicating that this combination of colors is characteristic of only oxLDL (Table 1).

### 2. Deposition of OxLDL in Excised Human Coronary Plaques Observed with CFM Incidence of OxLDL

**Figure 2:**
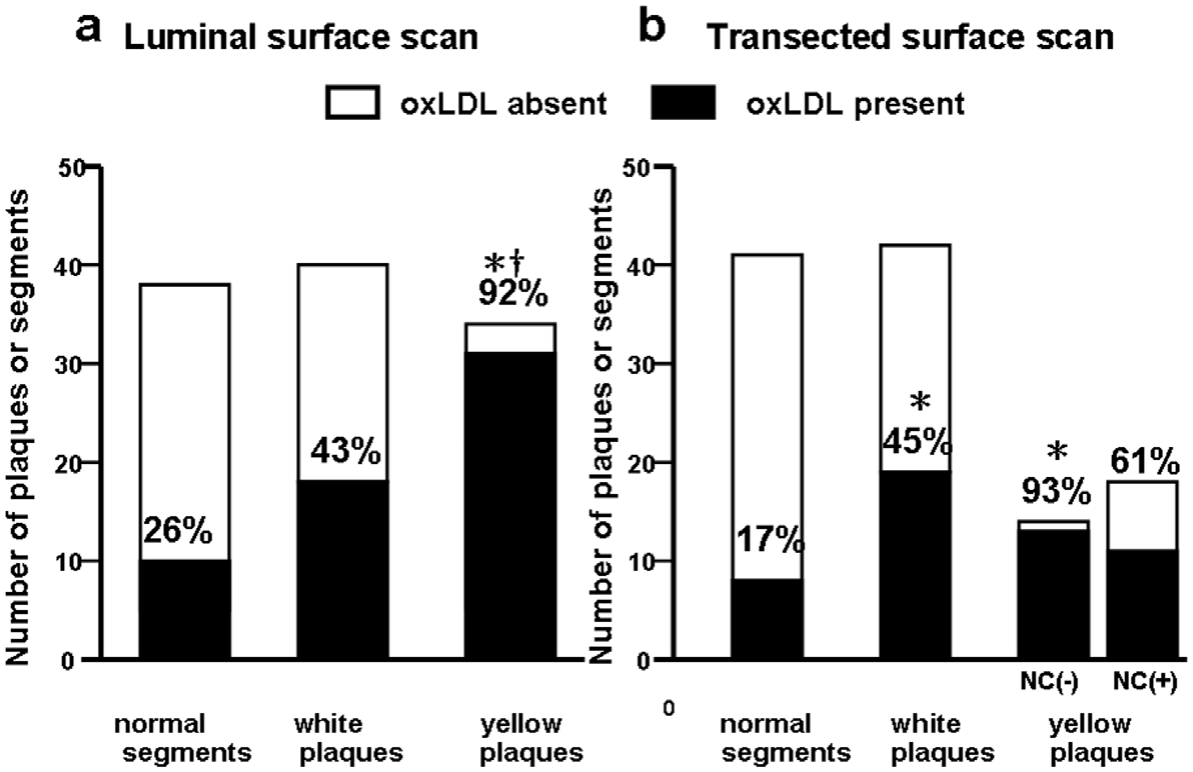
Percentage incidence of oxidized low-density lipoprotein (oxLDL) in human coronary arterial wall. Luminal surface scanning (a). The % incidence of oxLDL in yellow plaques was higher than those in normal segments and white plaques. *p<0.05 vs normal segments. ^†^p<0.05 vs white plaques. Transected surface scanning (b). The % incidence of oxLDL in white and yellow plaques without necrotic core [NC(−)] was higher than that in normal segments while that in yellow plaques with necrotic core [NC(+)] not. Percentage in each column indicates incidence of oxLDL.

On scanning the luminal surface, the % incidence of oxLDL deposition in 38 normal segments, 41 white plaques and 32 yellow plaques was 26, 44 and 94, respectively, indicating higher (p<0.05) incidence of oxLDL in yellow plaques than normal segment and white plaques. (Fig. 2A).

On scanning the transected surface, the % incidence of oxLDL deposition in 41 normal segments, 42 white plaques, 14 yellow plaques without necrotic core (NC), and 18 yellow plaques with NC was 17, 45, 93 and 61, respectively, indicating higher incidence of oxLDL in white plaques and yellow plaques without NC than normal segments. There was a tendency that the % incidence in yellow plaques with NC was lower than that of yellow plaques without (Fig. 2B).

### Deposition Patterns of OxLDL Studied by Luminal Surface Scanning

The location on the scanned surface that exhibited combined violet and reddish-brown fluorescence was considered to be the site of deposited oxLDL.

**Figure 3:**
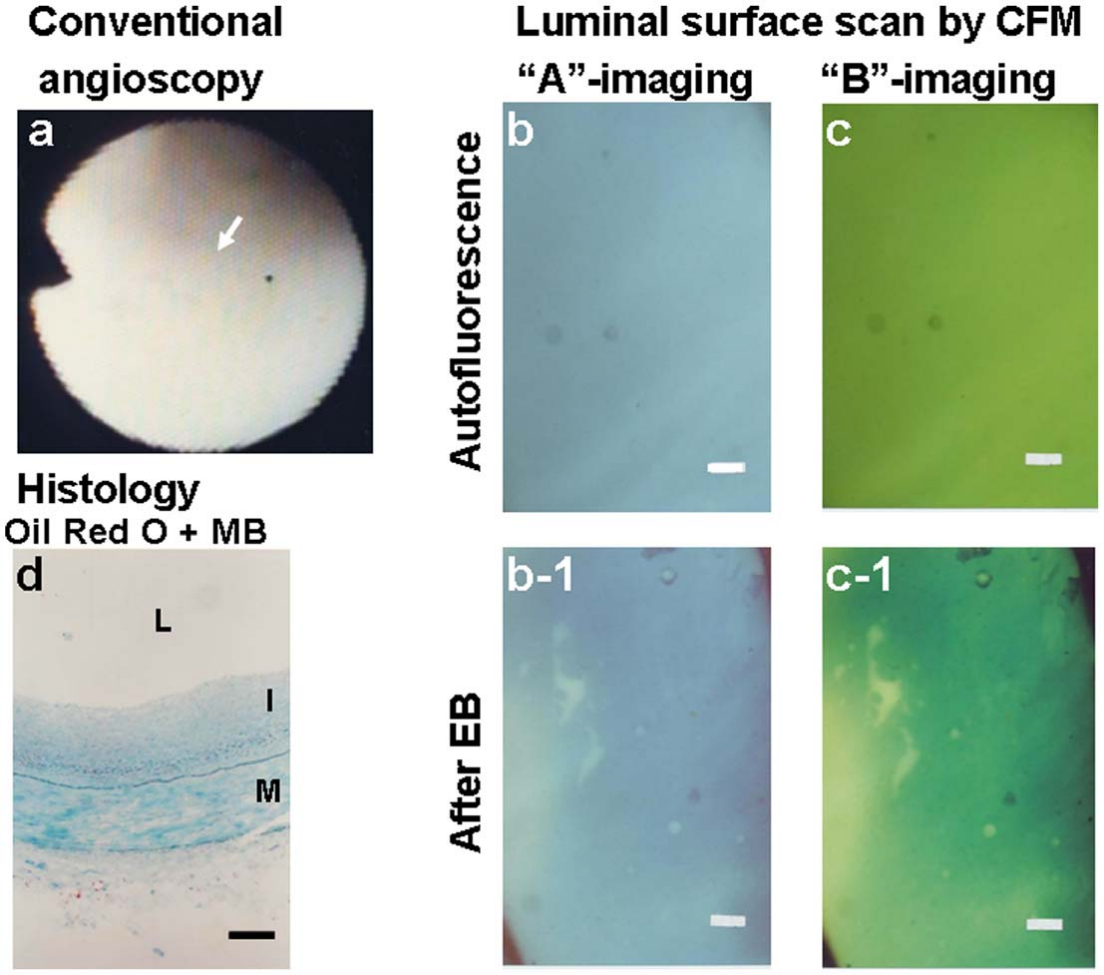
Luminal surface of a normal coronary segment without oxLDL. Scanning the luminal surface of a normal coronary segment from a 58-year old woman who died of hepatocellular carcinoma. Normal segment imaged by conventional angioscopy (a). Arrow indicates the portion observed by color fluorescence microscopy (CFM). (b, c) Images obtained by scanning the luminal surface of the same segment before administration of Evans blue (EB) dye. The plaque shows blue fluorescence with “A”-imaging and green fluorescence with “B”-imaging, indicating the existence of collagen I and absence of β-carotene that co-deposits with lipids [23], [24]. (b-1, c-1) Images of the same segment after administration of EB. The segment did not exhibit violet fluorescence with “A”-imaging or reddish-brown fluorescence with “B”-imaging, indicating the absence of oxLDL. (d) Histology of the same segment after Oil Red-O and methylene blue dye staining reveals the rich existence of collagen fibers (blue portions) in the intima but no deposition of lipids (red portions) or calcium compounds (black portions). L, I and M in this and the following figures: lumen, intima and media, respectively. Bars = 100-µm.

Figure 3 shows a normal segment by conventional angioscopy. Before administration of EB, the luminal surface exhibited blue and green fluorescence at “A”- and “B”-imaging, respectively, indicating presence of abundant collagen I. After administration of EB, the luminal surface exhibited dark green fluorescence at “B”-imaging, also indicating presence of collagen I but absence of oxLDL.

**Figure 4:**
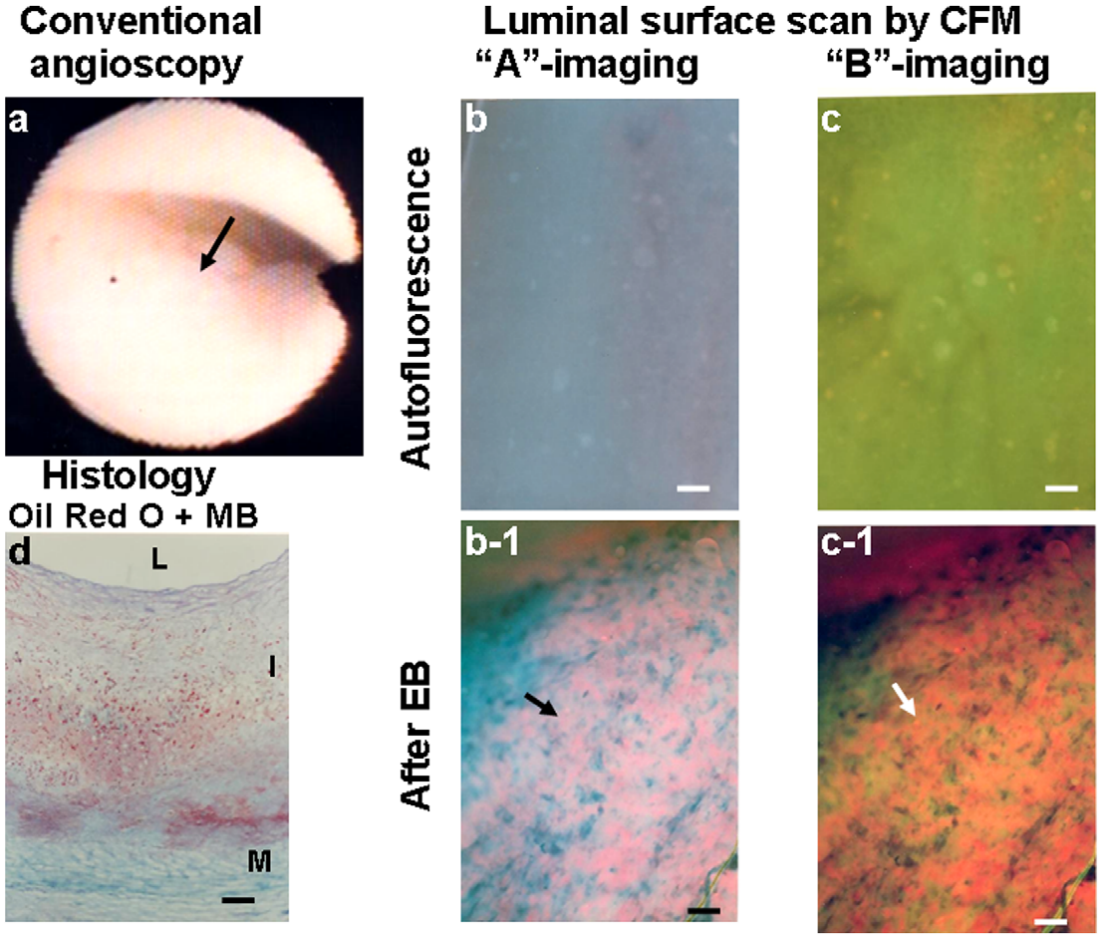
Luminal surface of a white coronary plaque with oxLDL. Scanning the luminal surface of a white plaque in a 61-year old man who died of chronic kidney disease. White plaque imaged by conventional angioscopy (a). Before administration of EB, scanning the luminal surface of the portion indicated by the arrow in (a) showed blue fluorescence with “A”-imaging (b) and green fluorescence with “B”-imaging (c), indicating abundant collagen I. After administration of EB, the same luminal surface exhibited diffuse violet fluorescence with “A”-imaging (arrow in b-1) and reddish-brown fluorescence with “B”-imaging (arrow in c-1), indicating deposition of oxLDL. Histologically, the intima was thick and a small amount of lipids was deposited in the deep layer but not in the superficial layer (d).

Figure 4 shows a yellow plaque by conventional angioscopy. Although the plaque exhibited blue and green fluorescence respectively at “A”- and “B”-imaging before, after the administration of EB the plaque exhibited diffuse violet and reddish-brown fluorescence at “A”- and “B”-imaging, indicating diffuse deposition of oxLDL.

**Figure 5:**
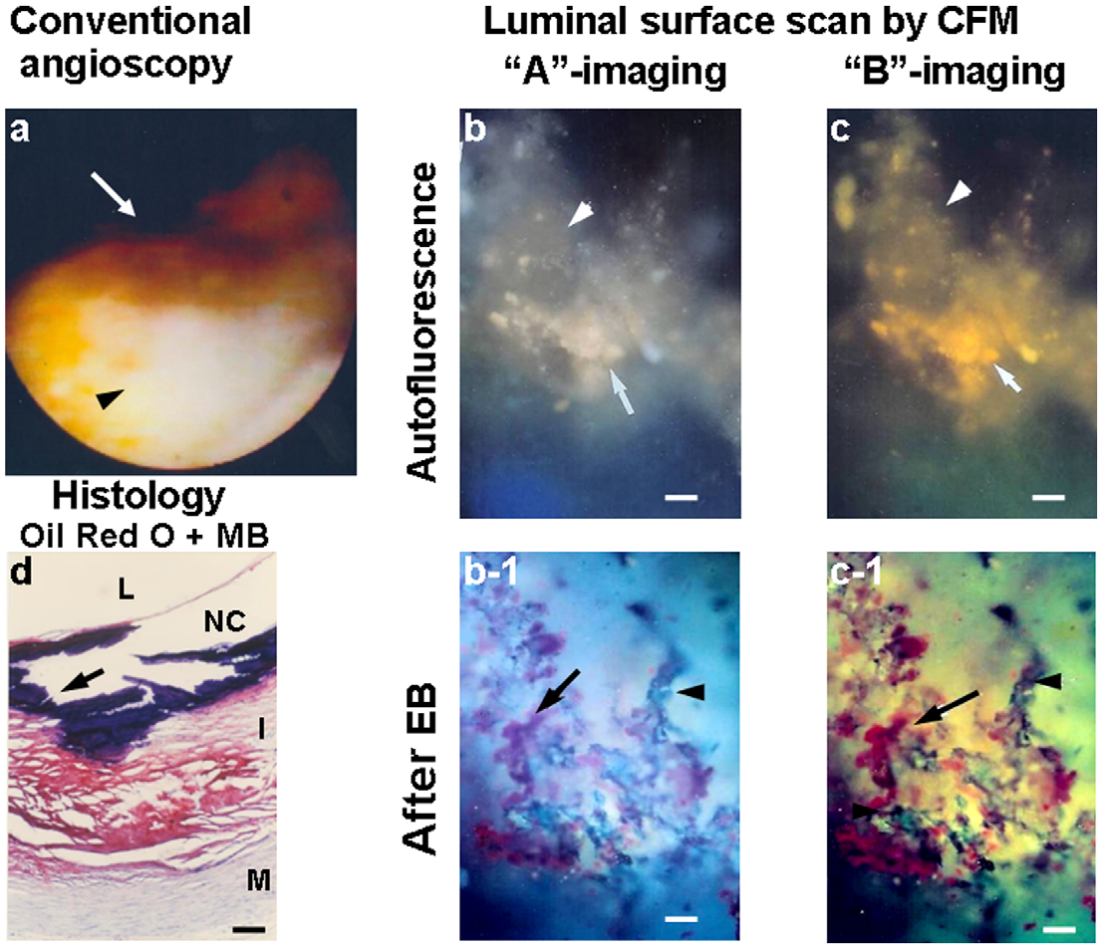
Luminal surface of a yellow coronary plaque with oxLDL. Scanning the luminal surface of a yellow plaque from a 66-year-old man who died of acute myocardial infarction. Yellow plaque with a white patch (arrow in a), suggesting co-deposition of lipids, β-carotene and calcium compound. Before administration of EB, scanning the transected surface of this plaque showed solid (arrow in b) and amorphous white-to-light yellow substances with “A”-imaging (arrowhead in b) and solid (arrow in c) and amorphous yellow-to-orange substances (arrowhead in c) with “B”-imaging, suggesting deposition of crystallized and amorphous calcium compounds and/or cholesterol (Table 1). After administration of EB, web-like and violet (arrow in b-1) and reddish-brown substances (arrow in c-1) appeared, indicating deposition of oxLDL. Arrowheads in b-1 and c-1 show collagen I. Histology revealed a thin fibrous cap with a necrotic core (NC) and calcium compounds (arrow in d) deposited underneath. The contents of the NC were lost during staining.

Figure 5 shows a dark yellow plaque by conventional angioscopy with a NC by histology. Before administration of EB, different to Figures 3 and 4, solid matters exhibiting white and yellow fluorescence respectively at “A”- and “B”-imaging were observed, indicating deposition of calcium compound but absence of collagen I (see Table 1). After the administration of EB, violet and reddish-brown fluorescence with web-like distribution was elicited respectively at “A”- and “B”-imaging, indicating web-like deposition of oxLDL.

Thus, deposition pattern was classified as patchy, diffuse and web-like. Although the former two deposition patterns were observed in normal segments, white plaques and yellow plaques, web-like pattern was observed in yellow plaques but not in other groups (Table 2).

**Table 2:** Deposition Patterns of Oxidized Low-density Lipoprotein (OxLDL) Visualized by Luminal Surface Scan by Color Fluorescent Microscopy (CFM).

		Normal segments	White plaques	Yellow plaques
n		38	41	32
Patchy deposition		5	5	5
(%)		13	12	14
Diffuse deposition		5	13	14
(%)		13	32	44
Web-like deposition		0	0	11* †
(%)		0	0	34

n = number of preparations examined.

* p<0.05 vs normal segments.

† p<0.05 vs white plaques.

### Deposition Sites of OxLDL Studied by Scanning the Transected Surface

**Figure 6:**
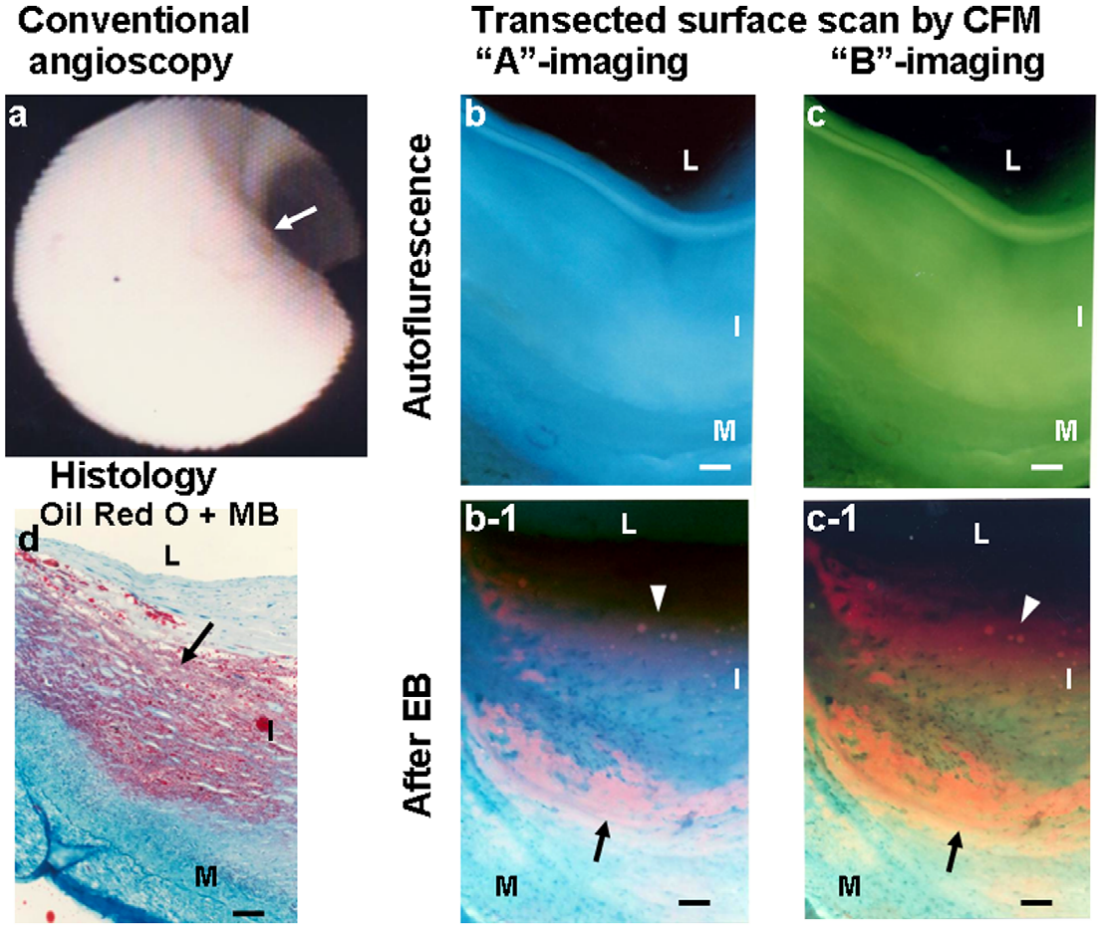
Transected surface of a white coronary plaque with oxLDL. Scanning the transected surface of a white plaque from a 56-year-old woman who died of subarachnoid hemorrhage. White plaque observed by conventional angioscopy (a). Before administration of Evans blue (EB) dye, the plaque indicated by an arrow in (a) exhibited blue fluorescence with “A”-imaging (b) and green fluorescence with “B”-imaging (c). After administration of EB, violet fluorescence with “A”-imaging and reddish-brown fluorescence with “B”-imaging were observed in the superficial [arrowheads in (b-1) and (c-1)] and deep layer [arrows in (b-1) and (c-1)], indicating deposition of oxLDL. Histology revealed collagen fiber-rich plaque with lipid deposition in the deep layer (d).

Figure 6 shows a white plaque in which oxLDL was deposited in both the superficial and deep layers.

**Figure 7:**
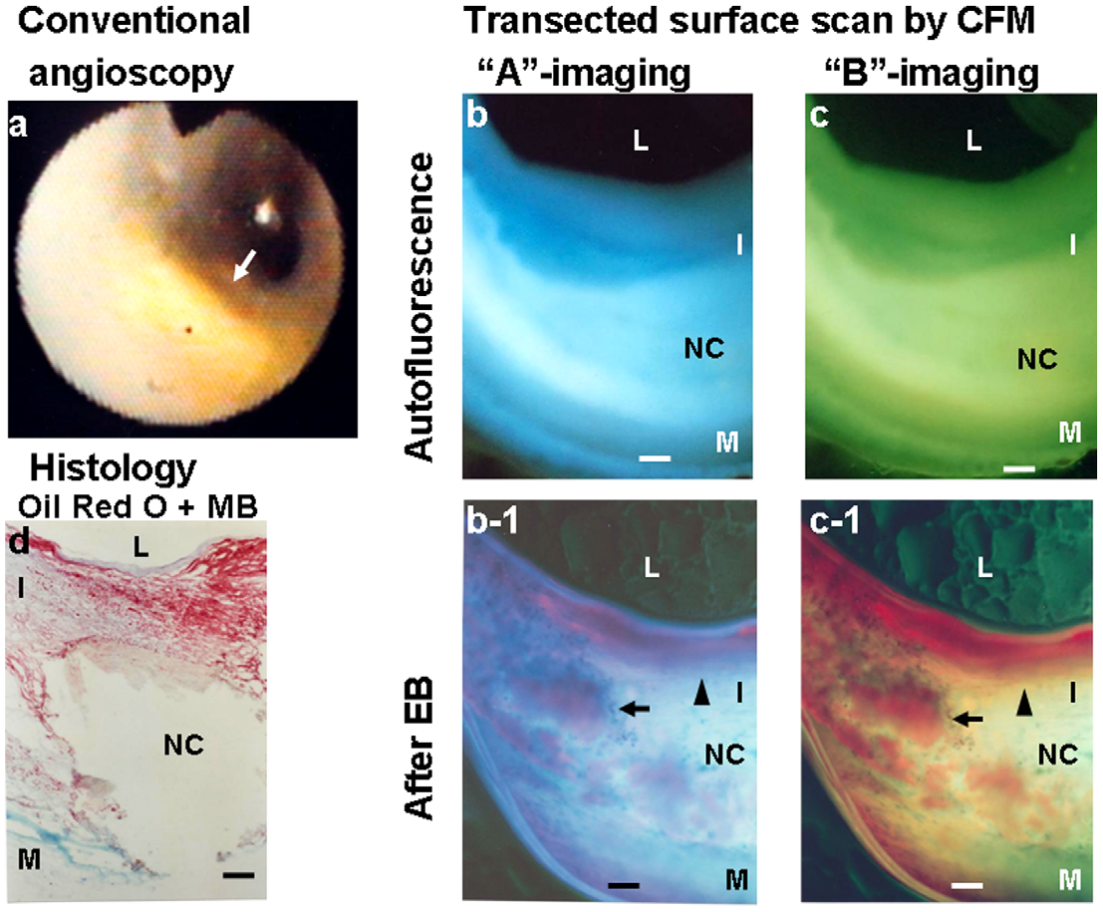
Transected surface of a yellow coronary plaque with oxLDL. Scanning the transected surface of a yellow plaque with necrotic core from a 60-year-old man who died of acute myocardial infarction. Yellow plaque observed by conventional angioscopy (arrow in a). Before administration of EB, the plaque showed a white-to-light blue layer covered by a blue layer with “A”-imaging (b) and a white-to-light green layers with “B”-imaging, suggesting the presence of a NC covered by fibrous cap. After administration of EB, violet fluorescence with “A”-imaging and reddish-brown fluorescence with “B”-imaging were observed in the fibrous cap (arrowheads in b-1 and c-1) and within the NC (arrows in b-1 and c-1), indicating deposition of oxLDL not only in the fibrous cap but also in the NC. NC was confirmed by histology (d).

Figure 7 shows a glistening yellow plaque. This plaque had a NC and oxLDL deposited marginal zones of the NC and also within the thin fibrous cap.

**Figure 8:**
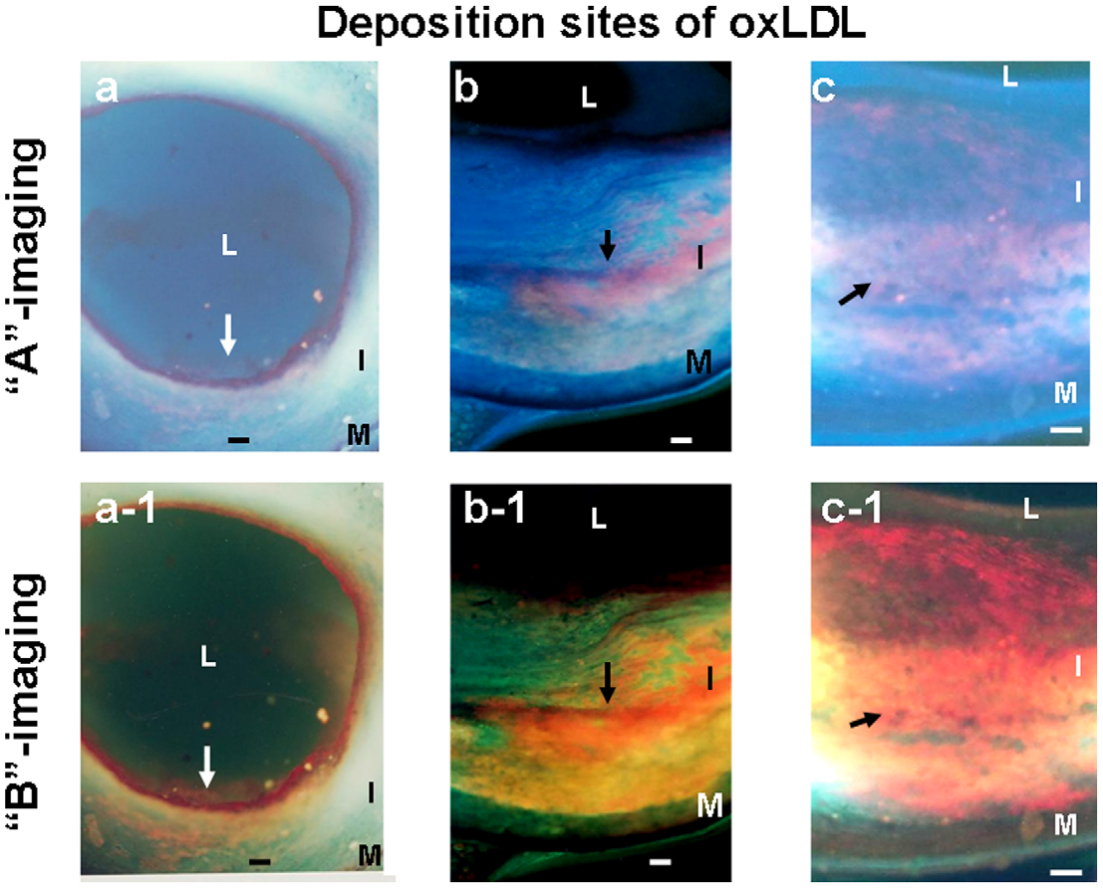
Deposition patterns of oxLDL observed by color fluorescent microscopy (CFM). Scanning the transected surface for the deposition patterns of oxLDL. Three patterns of deposition were observed with color fluorescence microscopy (CFM): superficial layer deposition (arrows in a and a-1), deep layer deposition (arrows in b and b-1), and deposition in both layers (arrows in c and c-1).

As shown in Figure 8, oxLDL deposited either in superficial layer (a, a-1), deep layer (b, b-1), or in both layers (c, c-1).

There was no significant difference in the incidence of oxLDL between yellow plaques with NC and those without. Deposition in superficial layer was predominantly observed in normal segments, deep layer deposition was not different among the three groups. Deposition in both layers in white plaques and yellow plaques with or without NC was significantly higher than normal segments (Table 3). These data indicate extensive oxLDL deposition in white and yellow plaques and localized deposition in normal segments.

**Table 3:** Deposition Sites of Oxidized Low-density Lipoprotein (OxLDL) Visualized by Transected Surface Scan by Color Fluorescent Microscopy (CFM).

	Normal segments	White plaques	Yellow plaques	
			NC(−)	NC(+)
**N**	41	42	14	18
**OxLDL visualized**	7	19*	13*	11
**(%)**	17	45	93	61
**Superficial layer deposition**	6	3	0	1
**(%)**	85	15	0	0
**Deep layer deposition**	1	2	1	0
**(%)**	14	11	8	0
**Deposition in both layers**	0	14	12	10
**(%)**	0	73† ‡	92† ‡	91† ‡

n = number of preparations examined. NC(−) : necrotic core absent. NC(+): necrotic core present.

* p<0.05 vs normal segments.

† p<0.05 vs superficial layer deposition.

‡ p<0.05 vs deep layer deposition.

### 3. Relationship Between Deposition of OxLDL and Lipids Stained by Oil Red O and Methylene Blue Dyes

**Figure 9:**
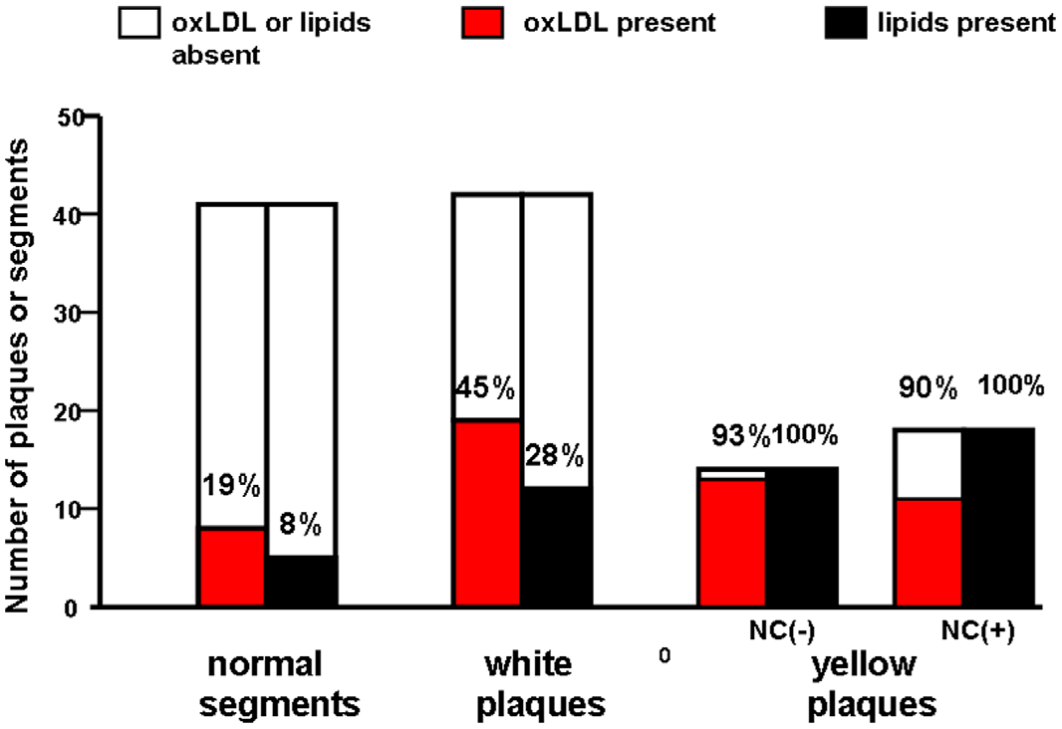
Co-deposition of oxidized low-density lipoprotein (oxLDL) with other lipid components that were stained red with Oil Red O and methylene blue dye (MB). Co-deposition of oxLDL and lipids was examined by transected surface scanning. Deposition of oxLDL without lipids was observed in several normal segments and white plaques, while deposition of lipids without oxLDL was observed in several yellow plaques, indicating that oxLDL did not necessarily co-deposit with lipids.

The lipids that are stained by Oil Red O are cholesterol and cholesteryl esters and ceramide (unpubl. obs.), so in the present study the substances that were stained red in the coronary specimens were considered to be these lipids. There were normal segments and white plaques in which oxLDL deposited but the lipids did not. The lipids deposited in all yellow plaques but there were plaques in which oxLDL did not, indicating that it did not necessarily co-deposit with the lipids (Fig. 9).

## Discussion

We investigated low-molecular-weight substances that do not autofluoresce but elicit fluorescence when conjugated to oxLDL and discovered that EB elicits a reddish-brown fluorescence that is specific for oxLDL with “B”-imaging. Consequently, we used it with CFA to image oxLDL in human coronary plaques in both in vitro and in vivo [25]. In a recent study, we also discovered that EB elicits violet fluorescence of oxLDL with “A”-imaging, so in the present study these two fluorescence colors in combination were used to increase the accuracy of identifying oxLDL.

The mechanism by which fluorescence of oxLDL is evoked is considered to be that EB deoxidizes oxLDL or that they conjugate each other to form an adduct eliciting the fluorescence colors. Because there are no other known major substances that show this combination of fluorescence colors, we considered this combination to be characteristic of oxLDL and that EB can be used as its biomarker.

### Localization of oxLDL and its Relation to Plaque Morphology

Localization of oxLDL has been studied mostly by immunohistochemical techniques; Kayo, et al observed deposition of oxLDL in NC (lipid core) but not in other portions of ruptured human coronary plaques that were obtained by endoatherectomy [19]; Fukuchi, et al observed 8-isoprostaglandin F (2 alpha), a marker of oxLDL, in deep layers of physiologically thickened intima of excised human coronary artery but did not examine this substance in plaques [20]. In the present study, different to these observations, oxLDL deposited not only in NC but also in other portions of the plaques, more frequently in yellow plaques without NC and white plaques, and frequently in superficial layers of normal coronary segments. Similar to our observation in normal coronary segments, Okura, et al observed deposition of oxLDL in superficial intima of early atherosclerotic lesions of human carotid, aortic and femoral arteries [30].

In the present study, the incidence of oxLDL increased in the order of normal segments, white plaques and yellow plaques without NC, but tended to decrease in yellow plaques with NC.

White plaques are formed by intimal thickening induced mainly by proliferated collagen fibers; the white plaques grow into yellow plaques by accumulating lipids in themselves; acquire NC; and finally become vulnerable to disruption [1], [31]. Therefore, the results in the present study indicate that oxLDL begins to deposit in the human coronary artery wall earlier than formation of plaques and increasingly deposits with plaque growth, but tend to decrease after NC formation.

Although oxLDL is considered to be essential for formation of yellow plaques with necrotic core, a small number of yellow plaques with necrotic core did not contain oxLDL in the present study. It is known that chemical changes occur in the oxLDL that is accumulated in macrophages [32], [33]. These changes might have been a reason for the absence of fluorescence colors characteristic of oxLDL in these yellow plaques.

Both white plaques and normal segments were classified as with or without oxLDL deposition. No obvious differences for this result were found by histology or the sex and age of the patient. Growth factors such as fibroblast growth factor-2 and osteoprotegerin [34] but not oxLDL are required for formation of white plaques because they are composed mainly of proliferated collagen fibers. It remains to be clarified what factor(s) determines initiation of oxLDL deposition.

### Deposition Patterns of oxLDL

Deposition patterns of native oxLDL in vascular wall, especially in human coronary arterial wall, has been unclear because of a lack in systematic examinations on them.

In the present study, the pattern of oxLDL deposition was classified as patchy, diffuse or web-like on scanning of the luminal surface. Deposition patterns were closely related to plaque morphology; in a patchy pattern in normal segments and white plaques, in a diffuse pattern in white plaques and yellow plaques without NC, and in web-like pattern in yellow plaques with NC. Although definite evidence is lacking, it is possible that the patchy and diffuse patterns indicate free deposition of oxLDL in the tissues, whereas the web-like deposition may indicate oxLDL accumulated in macrophages/foam cells, because this distribution pattern was observed solely in yellow plaques with a NC and because macrophages are known to exist in the fibrous cap covering the NC and accumulate oxLDL in themselves [7].

### Images by Other Techniques

In most reports on molecular imaging by spectroscopy, oxLDL and other substances in atherosclerotic lesions showed dot-like distribution patterns [15], [18]. In contrast, in this and our previous studies using CFA and CFM, dot-like deposition of lipoproteins, apolipoproteins, cholesterol or cholesterol esters was rare [25], [26], [35], [36]. The difference is possibly related to the cut-off of the low-intensity portions of the obtained images to enhance the high-intensity portions, leading to a dot-like distribution in spectroscopy.

### Clinical Application of EB as a Biomarker of oxLDL

There are many hard lines for clinical application of immunohistochemical techniques and radiolabelled antibodies or nanoparticles for imaging oxLDL in patients in vivo.

EB has been clinically used for staining fibrin and the damaged vascular endothelial cells for many years and its clinical safety has been proven [22]–[24], [37]. By using EB as a selective biomarker of oxLDL, we succeeded in imaging of oxLDL within coronary plaques by a CFA system in patients during coronary angiography [25]. However, imaging was limited to oxLDL deposited within 200-µm from coronary luminal surface and detailed evaluation of its distribution patterns was difficult because of low sensitivity and magnification power of this CFA system. By increasing sensitivity and magnification power, the localization and deposition patterns of oxLDL in coronary arterial wall can be evaluated in patients in vivo.

### Conclusion

By using EB as a biomarker, deposition sited and patterns of oxLDL within coronary plaques and normal coronary segments of humans was successfully imaged by CFM. The results suggest that oxLDL begins to deposit in the wall of the human coronary artery before plaque formation and increasingly deposits with plaque growth, but tends to decrease after NC formation.

### Study Limitations

The present experimental study of the sites and patterns of oxLDL deposition within human coronary plaques using CFM and EB as a biomarker seems to be the first of its kind. However, because atherosclerotic plaques contain a number of substances, there may be other unknown substances that exhibit the same fluorescence color in the presence of EB.
